# Assessing the Causal Effects of Adipokines on Uric Acid and Gout: A Two-Sample Mendelian Randomization Study

**DOI:** 10.3390/nu14051091

**Published:** 2022-03-05

**Authors:** Ruyi Cong, Xiaoyu Zhang, Zihong Song, Shanshan Chen, Guanhua Liu, Yizhi Liu, Xiuyu Pang, Fang Dong, Weijia Xing, Youxin Wang, Xizhu Xu

**Affiliations:** 1 School of Public Health, Shandong First Medical University & Shandong Academy of Medical Sciences, Tai’an 271000, China; rycong9832@163.com (R.C.); zhsong1996@163.com (Z.S.); cshanshan2022@163.com (S.C.); liuguanhua200210@163.com (G.L.); yzliu20080910@163.com (Y.L.); pangxy123@163.com (X.P.); fdong@sdfmu.edu.cn (F.D.); wjxing@sdfmu.edu.cn (W.X.); 2 Department of Anesthesiology, Sanbo Brain Hospital, Capital Medical University, Beijing 100093, China; hydzxy@126.com; 3 Beijing Key Laboratory of Clinical Epidemiology, School of Public Health, Capital Medical University, Beijing 100069, China; wangy@ccmu.edu.cn; 4 School of Medical and Health Sciences, Edith Cowan University, Perth 6027, Australia; 5 The Second Affiliated Hospital of Shandong First Medical University, Tai’an 271000, China

**Keywords:** gout, uric acid, adiponectin, soluble leptin receptors, mendelian randomization

## Abstract

Previous observational studies have highlighted associations between adipokines and hyperuricemia, as well as gout, but the causality and direction of these associations are not clear. Therefore, we attempted to assess whether there are causal effects of specific adipokines (such as adiponectin (ADP) and soluble leptin receptors (sOB-R)) on uric acid (UA) or gout in a two-sample Mendelian randomization (MR) analysis, based on summary statistics from large genome-wide association studies. The inverse-variance weighted (IVW) method was performed as the primary analysis. Sensitivity analyses (including MR-Egger regression, weighted median, penalized weighted median, and MR pleiotropy residual sum and outlier methods) were also performed, to ensure reliable results. In the IVW models, no causal effect was found for sOB-R (odds ratios (OR), 1.002; 95% confidence intervals (CI), 0.999–1.004; *p* = 0.274) on UA, or ADP (OR, 1.198; 95% CI, 0.865–1.659; *p* = 0.277) or sOB-R (OR, 0.988; 95% CI, 0.940–1.037; *p* = 0.616) on gout. The results were confirmed in sensitivity analyses. There was no notable directional pleiotropy or heterogeneity. This study suggests that these specific adipokines may not play causal roles in UA or gout development.

## 1. Introduction

Uric acid (UA) is a waste product of purine catabolism. It can lead to gout when this molecule nucleates in a joint or other tissue to form crystals of monosodium urate [1]. Gout is a chronic inflammatory disease that is influenced by genetic factors [2,3]. Gout and hyperuricemia have become a major risk factor to human health, which are caused by elevated UA [4]. Extensive evidence has shown that adipokines (such as adiponectin (ADP) and soluble leptin receptors (sOB-R)) may be associated with changes in UA levels and the development of gout [5,6,7,8,9]. Moreover, the causal relationship between ADP and UA concentrations was demonstrated by a Mendelian randomization (MR) study with Europeans [10].

Nevertheless, a large amount of inconsistent evidence was found in observational studies, suggesting that confounders and reverse causality may be involved in the casual effects of adipokines on UA and gout. The limitations of previous studies can be effectively addressed by using MR [11]. MR method is a genetic epidemiological method, which can assess causal inference by exploiting the genetic variants influencing a modifiable risk factor. According to Mendel’s genetic law, genetic material is randomly distributed during meiosis and passed from parents to offspring at conception. Therefore, it is less susceptible to residual confounding and reverse causality, and it makes causal inferences about the effect of exposure on outcomes, using genetic variations closely related to the exposure of interest as instrumental variables (IVs); which addresses the shortcomings of previous observational epidemiology.

We conducted this study based on a two-sample MR framework, to explore the causal effects of specific adipokines (i.e., ADP and sOB-R) on UA and gout.

## 2. Materials and Methods

This study is reported as per STROBE guidelines (Supplementary Table S1). In order for causality to be valid in MR analysis, the following three hypotheses must be satisfied: (a) The instruments of genetic variations must be robustly related to the concentration of adipokines; (b) The genetic variations must not be associated with any confounder of the adipokines and UA, as well as gout associations; And (c) the selected genetic variations should not affect the UA or gout independently of its effect on adipokines [12].

### 2.1. Datasource and Selection of Instruments for MR

#### 2.1.1. Outcome Datasource

Summary data for UA were obtained from a genome-wide association studies (GWAS) of 42,741 European participants within the Global Urate Genetics Consortium (GUGC) [13]. For gout, a large sample of 69,374 participants (2115 cases and 67,259 controls) were obtained from European populations within the GUGC [14]. We obtained these data information for analysis from published GWASs on 20 February 2022 (https://gwas.mrcieu.ac.uk/, accessed on 13 January 2022).

#### 2.1.2. Selection of Instruments for MR

Summary data for adipokine variants were obtained from published GWAS (https://gwas.mrcieu.ac.uk/, accessed on 8 September 2021) and publicly available GWAS databases on 20 February 2022 [15,16] (Supplementary Table S2). In this study, genetic variants were analyzed using MR, based on a significant genome-wide correlation with adipokine concentrations (i.e., the inclusion criteria of *p* value at <5 × 10^−8^). All of the variants were employed in linkage disequilibrium below 10%. Since this study only used publicly available summary statistic from relevant GWAS and did not use the individual data, ethical approval was not required.

For the causal effects of ADP (*n* = 39,883, individuals of mixed ethnicity (predominantly European)) on UA or gout, we selected two sets of IVs using 23 and 25 variants. In addition, rs2980879 and rs8060301 were removed, due to being palindromic with intermediate allele frequencies. 

Moreover, 4 variants locating in the *LEPR* gene were used to explain the causal effects of sOB-R (*n* = 1000 individuals of European ancestry) on UA or gout. Information on all single nucleotide polymorphisms (SNPs) as IVs involved in the MR analyses was provided in the supplementary materials (Supplementary Table S3).

#### 2.1.3. Statistics Power and F-Statistics

The power of this study was calculated using an online computing tool (https://shiny.cnsgenomics.com/mRnd/, accessed on 13 January 2022). We fixed the type-I error rate at 0.05 and the *R*^2^ of 0.05 for ADP and 0.001 for sOB-R, our study had sufficient power (>80%) to detect the effects of adipokines on UA or gout [16]. Furthermore, based on the approximation method, we calculated the mean F-statistic for each of the IVs selected [17]. 

### 2.2. Statistics Analysis

The standard inverse variance weighting (IVW) method assumed that each variant contained was a valid IV, and this was a standard MR method for summary data [17]. We used the IVW method as the primary analysis. The random effects IVW model was used by default, and the fixed effects model was used when the causal estimates between SNPs were under-dispersion [18]. In addition, in order for causality to be valid in MR analysis, we also performed a series of sensitivity analyses (such as weighted median, penalized weighted median, leave-one-out method, MR pleiotropy residual sum and outlier (MR-PRESSO); and MR-Egger regression) to test the robustness of the association. 

Weighted median was used to account for the estimators, even though up to 50% of the information was provided by invalid IVs [19]. Ineffective IV instruments affected the median estimate, even if they did not directly contribute to the median estimate; therefore, we also used penalized weighted median estimators [19]. In order to test the influence of each SNP on the results, we adopted the leave-one-out method, which was based on IVW point estimation after removing a SNP from the population [20]. The fluctuation of the results before and after removal reflected the sensitivity of this SNP [20]. MR-PRESSO consisted of three components (MR-PRESSO global test, MR-PRESSO outlier test, MR-PRESSO distortion test) and relied on a regression framework with regressions based on the effect of exposure on results provided by the slope of the regression line [21]. We also used MR-PRESSO to evaluate the extent of horizontal pleiotropy.

MR-Egger regression was used to examine the influence of pleiotropy, assuming that its strengths as an instrument did not affect the magnitude of the pleiotropic effects [17]. Estimates of the average pleiotropic effect of genetic variants included in the analysis could be explained by the MR-Egger intercept [22]. To quantify the heterogeneity of the selected variants, additionally, we assessed the Cochran’s *Q* statistic, which followed a distribution with χ^2^ degrees of freedom equal to the number of SNPs minus one [23]. 

The MR results were presented as odds ratios (OR) with 95% confidence intervals (CI) for each gene predicting increased risk factors. The link between exposure and outcome with a *p* value < 0.05 was a considered significant statistical difference. Packages (such as ‘TwoSampleMR’ (version 0.5.6) and ‘MR-PRESSO’ (version 1.0)) in Rstudio (R version 4.1.2, R Project for Statistical Computing) were used to perform MR analyses. 

## 3. Results

**Figure 1:**
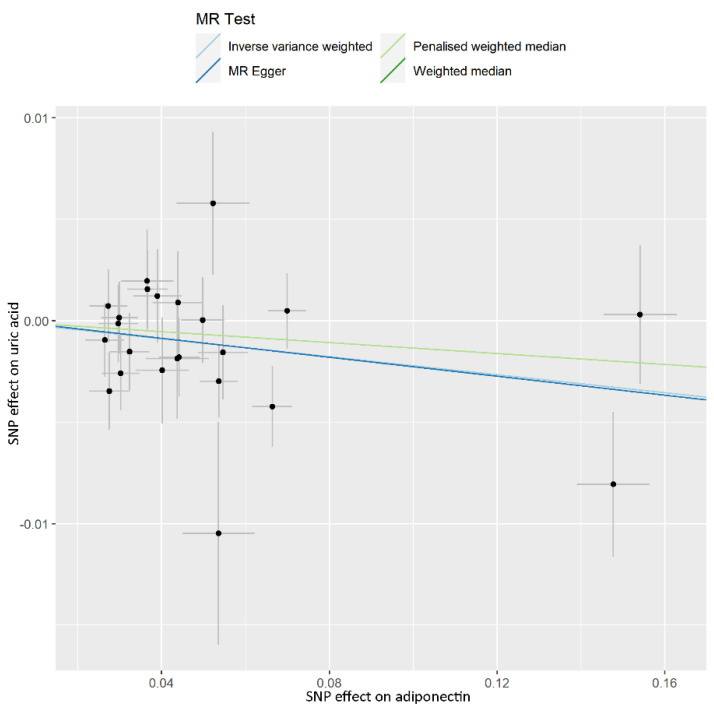
Scatter plot showing the associations of the SNP effects on the adiponectin, against the SNP effects on the uric acid. Circles indicate marginal genetic associations with adiponectin and risk of gout for each variant. Error bars indicate 95% CIs. MR: mendelian randomization; IVW: inverse-variance weighted; SNP: single nucleotide polymorphism.

As positive control, the causal effect of ADP on UA was confirmed in the IVW model (OR per 1 mg/dL decreased in ADP concentration: 0.978; 95% CI, 0.961–0.996; *p* = 0.016), although invalid results were found for the weighted median (OR, 0.987; 95% CI, 0.961–1.013; *p* = 0.324), penalized weighted median (OR, 0.987; 95% CI, 0.961–1.013; *p* = 0.311), and MR-Egger (OR, 0.977; 95% CI, 0.939–1.016; *p* = 0.256) analyses (Table 1). The estimated effect sizes of the SNPs on both the ADP and UA outcomes were displayed in a scatter plot (Figure 1). Leave-one-out analysis showed that the elimination of any SNP did not cause a change in the results (Supplementary Figure S1). Horizontal pleiotropy was not found using the MR-PRESSO global test (*p* = 0.438). The MR-Egger analysis (intercept = 0.00007; *p* = 0.946) also indicated that there was no notable directional pleiotropy. No heterogeneity for the selected variants was found using Cochran’s *Q* statistic (*p* = 0.389). The F-statistics of all 23 IVs were greater than 10 (Supplementary Table S3).

**Figure 2:**
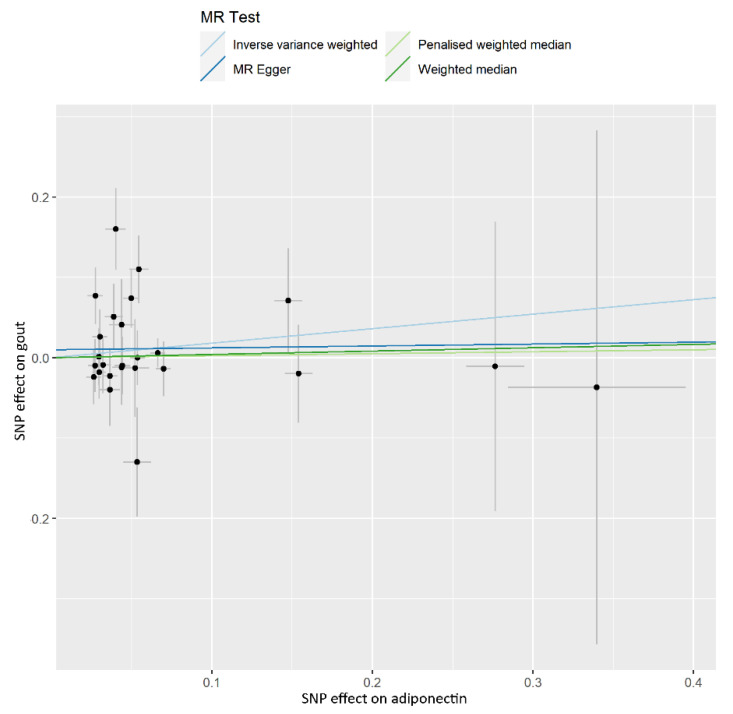
Scatter plot showing the associations of the SNP effects on adiponectin, against the SNP effects on gout. Circles indicate marginal genetic associations with adiponectin and risk of gout for each variant. Error bars indicate 95% CIs. MR: mendelian randomization; IVW: inverse-variance weighted; SNP: single nucleotide polymorphism.

In the IVW model, ADP concentration was unrelated to risk of gout (OR, 1.198; 95% CI, 0.865–1.659; *p* = 0.277), and similar invalid results were found for the weighted median (OR, 1.043; 95% CI, 0.698–1.556; *p* = 0.839), penalized weighted median (OR, 1.025; 95% CI, 0.692–1.519; *p* = 0.901), and MR-Egger (OR, 1.024; 95% CI, 0.513–2.045; *p* = 0.947) analyses (Table 1). The estimated effect sizes of the SNPs on both the ADP and gout outcomes were displayed in a scatter plot (Figure 2). The leave-one-out analysis showed that the elimination of any SNP did not cause a change in the results (Supplementary Figure S2). No horizontal pleiotropy was found using the MR-PRESSO global test (*p* = 0.116). The MR-Egger analysis (intercept = 0.024; *p* = 0.947) also indicated that there was no notable directional pleiotropy. No heterogeneity for the selected variants was found by using Cochran’s *Q* statistic (*p* = 0.083). The mean F-statistics were greater than 10 (Supplementary Table S3).

**Figure 3:**
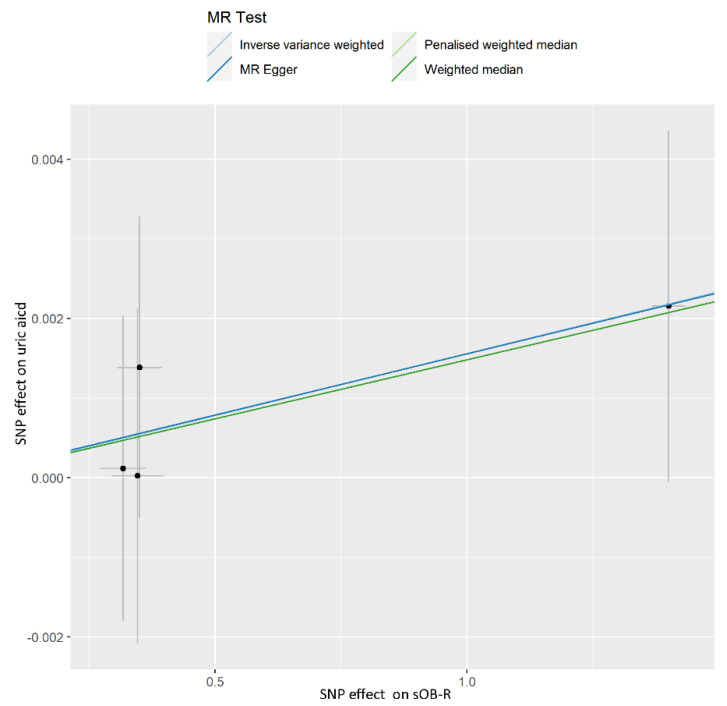
Scatter plot showing the associations of the SNP effects on the sOB-R against the SNP effects on the uric acid. Circles indicate marginal genetic associations with sOB-R and risk of uric acid for each variant. Error bars indicate 95% CIs. sOB-R: soluble leptin receptors; MR: mendelian randomization; IVW: inverse-variance weighted; SNP: single nucleotide polymorphism.

A causal effect of sOB-R on UA was not observed in our analysis. The invalid results were found for the IVW model (OR, 1.002; 95% CI, 0.999–1.004; *p* = 0.274), weighted median (OR, 1.001; 95% CI, 0.999–1.004; *p* = 0.326), penalized weighted median (OR, 1.001; 95% CI, 0.999–1.004; *p* = 0.325), and MR-Egger (OR, 1.002; 95% CI, 0.997–1.006; *p* = 0.578) analyses (Table 2). The results of the evaluation for each SNP were shown in the scatter plot (Figure 3). It was found that the deletion of a SNP did not cause any changes in the results through leave-one out sensitivity analysis (Supplementary Figure S3). The MR-PRESSO global test (*p* = 0.969) and MR-Egger analysis (intercept = 0.00002; *p* = 0.991) for UA showed that there was no horizontal pleiotropy. The result of Cochran’s *Q* statistic (*p* = 0.961) showed no heterogeneity among the selected variants. The F-statistics of all IVs were greater than 10 (Supplementary Table S3).

**Figure 4:**
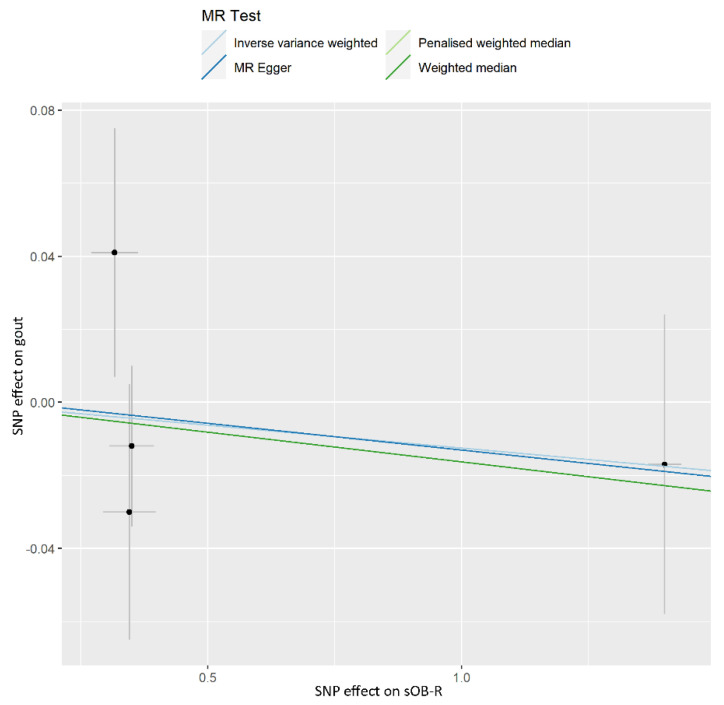
Scatter plot showing the associations of the SNP effects on sOB-R against the SNP effects on gout. Circles indicate marginal genetic associations with sOB-R and risk of gout for each variant. Error bars indicate 95% CIs. sOB-R: soluble leptin receptors; MR: mendelian randomization; IVW: inverse-variance weighted; SNP: single nucleotide polymorphism.

No evidence of a causal effect of sOB-R on gout was found with either model (such as the IVW model (OR, 0.988; 95% CI, 0.940–1.037; *p* = 0.616), weighted median (OR, 0.984; 95% CI, 0.933–1.037; *p* = 0.547), penalized weighted median (OR, 0.984; 95% CI, 0.933–1.037; *p* = 0.544), and MR-Egger (OR, 0.985; 95% CI, 0.901–1.078; *p* = 0.779)) (Table 2). The scatter plot showed the results of MR analysis of each SNP using the IVW model (Figure 4). SNP removal was found to not affect the results in the leave-one-out analysis (Supplementary Figure S4). No horizontal pleiotropy was found using the MR-PRESSO global test (*p* = 0.697) and MR-Egger analysis (intercept = 0.002; *p* = 0.959) for gout; and no heterogeneity was found for the selected variants using Cochran’s *Q* statistic (*p* = 0.492). The F-statistics of all IVs were greater than 10 (Supplementary Table S3).

## 4. Discussion

To our knowledge, this is the first attempt to explore the causal effects of ADP on gout, and sOB-R on UA and gout, based on MR analyses. Based on summary statistics from GWASs, we found no evidence to support the causal effects of these adipokines on UA or gout. These results were consistent across the sensitivity analyses using different methods.

ADP is the most abundant adipokine and is negatively correlated with adiposity. Extensive evidence has shown that obesity may be associated with higher UA levels and a greater risk of gout [24,25,26], and that ADP may play an important role. Numerous observational studies have investigated the relationship of circulating ADP and UA, with inverse associations generally observed. Decreased ADP levels lead to higher UA levels [6,10]. Our study reinforces this epidemiological evidence, by replicating the causal effect of ADP on UA. A study of gout in Japan reported that the role of ADP in gout was similar to that of UA [27]. Another study, however, reported that higher ADP concentrations in patients with severe gout compared to controls [28]. In our MR analysis, we found no causal effect of ADP on gout using a mixed-ethnicity sample. The two-sample MR analysis model, using genetic variations significantly associated with ADP as IVs, is not susceptible to confounding factors and reverse causality compared with observational studies.

Febuxostat, used for the treatment of hyperuricemia in gout, is a non-purine xanthine oxidase inhibitor [29]. In the course of treatment, the concentration of ADP was elevated, possibly due to the involvement of reactive oxygen species [30]. Similar results were seen in patients tread with benzbromarone for gout [31]. Benzbromarone is a potent UA excretion drug that works by inhibiting urate transporter 1 (URAT1) and glucose transporter 9 (GLUT9) [29]. URAT1A and GLUT9 are molecules expressed in proximal renal tubules that mediate renal reabsorption of UA [32,33,34,35]. Moreover, the increase of ADP concentration may not be associated with the decrease of UA, but benzbromarone-induced peroxisome proliferator-activated receptors (PPAR) α activation increased the mRNA of ADP via the promoter of ADP [36]. The increase in PPAR γ mRNA induced by benzbromarone may play a role in the mRNA expression of ADP in 3T3L1 cells [36]. Therefore, we speculate that drug therapy may be an important factor in the elevation of ADP in patients with gout. In addition, insulin resistance leads to increases in URAT1 and GLUT9 [29]. Under a high purine load, insulin resistance can enhance UA reabsorption, which is manifested as upregulation of URAT1 expression [37]. Similarly, elevated levels of URAT1 protein have been observed in obesity/metabolic syndrome model mice [38]. In obese patients, ADP is thought to be closely related to insulin resistance [7]. Overall, the correlation between ADP and gout in observational studies may only be an accompanying relationship or influenced by confounding factors, and ADP does not play a direct role in the occurrence and development of gout.

As the main leptin receptor in circulating blood, sOB-R is closely related to leptin binding activity [39]. Numerous studies have shown that high levels of UA are accompanied by high levels of serum leptin [40,41,42,43]. High concentrations of leptin were detected in both severe gout patients and in the acute phase of gout [8,27]. Under the action of a feedback regulation mechanism, the concentration of sOB-R in circulating blood may be influenced by changes in leptin levels. A study suggested that the mRNA levels of leptin receptors in peripheral blood mononuclear cells of patients with gout were significantly elevated, usually binding to leptin and transducing downstream signals [8]. We also attempted to investigate the causal effects of leptin on UA and gout, but there were not enough SNPs as IVs to support MR analyses. Based on a two-sample MR framework and using a large European population sample, we also found no evidence of a causal effect of sOB-R on UA or gout. 

Studies have shown that females have significantly higher leptin levels than males, possibly due to the testosterone effect in men, which inhibits leptin production in adipocytes [41,42,43,44,45,46]. Leptin concentrations were positively correlated with UA in premenopausal females and elevated in females with hyperuricemia [47,48]. Therefore, the concentration of sOB-R, which is closely related to leptin levels, may also differ between the sexes. Similarly, UA differs between the sexes and is thought to be caused by estrogen [49,50]. This phenomenon suggests that estrogen and the over-representation of females in the sample may have influenced these results. However, these results may be related to the fact that gender-specific adipokines data are not available. Therefore, the causal effects of adipokines on UA cannot be accurately described between the sexes. This limitation reflects the need for sex-stratified GWASs and sex-specific research in this area of the causal effects of adipokines on UA and gout in the future.

There are three main strengths of our MR analyses in the present study. First of all, we used large-scale summary data sets of ADP, sOB-R, gout, and UA. Second, we found no heterogeneity or pleiotropic of the IVs using multiple sensitivity analysis models and Cochran’s *Q* statistic. Thus, despite the limited number of powerful genetic instruments, the accuracy of the resulting MR estimates and the reliability of the results were significantly improved. Third, our MR analyses more effectively avoided potential confounding factors and reverse causality than traditional observational studies.

Without doubt, there were several limitations to our analyses. First, in terms of data, we selected a mixed population sample of ADP, and the resulting racial differences should not be ignored. Next, summary level data were used in MR analyses, so it was not possible to stratify the analysis by covariates of interest. Finally, our samples were principally European, which restricts the universality of our results to other ethnic groups.

## 5. Conclusions

In summary, in this two-sample MR study, our results do not support causal effects of these specific adipokines on UA or gout. Our results suggest that these specific adipokines do not play a causal role in UA or gout development.

## Figures and Tables

**Table 1:** Causal effects of ADP on UA and gout using MR analyses.

Phenotype	Numbers of SNPs	OR (95% CI)	Beta (SE)	*p*	Q Statistic	F-Statistic
ADP vs. UA						4349.6
IVW	23	0.978 (0.961–0.996)	−0.022 (0.009)	0.016	0.389	
Weighted median	23	0.987 (0.961–1.013)	−0.013 (0.014)	0.324		
Penalised weighted median	23	0.987 (0.961–1.013)	−0.013 (0.201)	0.311		
MR-PRESSO	23		−0.017 (0.011)	0.146		
global test				0.438		
MR-Egger	23	0.977 (0.939–1.016)	−0.023 (0.020)	0.256		
egger_intercept			0.00007 (0.001)	0.946		
ADP vs. gout						5751.4
IVW	25	1.198 (0.865–1.659)	0.181 (0.166)	0.277	0.083	
Weighted median	25	1.043 (0.698–1.556)	0.042 (0.204)	0.839		
Penalised weighted median	25	1.025 (0.692–1.519)	0.025 (0.201)	0.901		
MR-PRESSO	25		0.181 (0.166)	0.288		
global test				0.116		
MR-Egger	25	1.024 (0.513–2.045)	0.024 (0.353)	0.947		
egger_intercept			0.010 (0.019)	0.618		

Beta is the estimated effect size. *p* < 0.05 was considered statistically significant. ADP: adiponectin; CI: confidence intervals; IVs: instrumental variables; IVW: inverse-variance weighted; MR: mendelian randomization; MR-PRESSO: pleiotropy residual sum and outlier; OR: odds ratio; SE: standard error; SNP: single-nucleotide polymorphism.

**Table 2:** Causal effects of sOB-R on UA and gout using MR analyses.

Phenotype	Numbers of SNPs	OR (95% CI)	Beta (SE)	*p*	Q Statistic	F-Statistic
sOB-R vs. UA						44.8
IVW	4	1.002 (0.999–1.004)	0.002 (0.001)	0.274	0.961	
Weighted median	4	1.001 (0.999–1.004)	0.001 (0.002)	0.326		
Penalised weighted median	4	1.001 (0.999–1.004)	0.001 (0.002)	0.325		
MR-PRESSO	4		0.002 (0.0004)	0.040		
global test				0.969		
MR-Egger	4	1.002 (0.997–1.006)	0.002 (0.002)	0.578		
egger_intercept			0.00002 (0.002)	0.991		
sOB-R vs. gout						71.4
IVW	4	0.988 (0.940–1.037)	−0.013 (0.025)	0.616	0.492	
Weighted median	4	0.984 (0.933–1.037)	−0.016 (0.027)	0.547		
Penalised weighted median	4	0.984 (0.933–1.037)	−0.016 (0.027)	0.544		
MR-PRESSO	4		−0.013 (0.022)	0.615		
global test				0.697		
MR-Egger	4	0.985 (0.901–1.078)	−0.015 (0.046)	0.779		
egger_intercept			0.002 (0.028)	0.959		

Beta is the estimated effect size. *p* < 0.05 was considered statistically significant. sOB-R: soluble leptin receptors; UA: uric acid; CI: confidence intervals; IVs: instrumental variables; IVW: inverse-variance weighted; MR: mendelian randomization; MR-PRESSO: pleiotropy residual sum and outlier; OR: odds ratio; SE: standard error; SNP: single-nucleotide polymorphism.

## Data Availability

The data presented in this study are available in published genome-wide association studies (GWAS) (https://gwas.mrcieu.ac.uk/, accessed on 13 January 2022).

## Supplementary Materials

The following supporting information can be downloaded at: https://www.mdpi.com/article/10.3390/nu14051091/s1, Figure S1. Leave-one-out analysis of the effect of the adiponectin on the uric acid; Figure S2. Leave-one-out analysis of the effect of the adiponectin on the gout; Figure S3. Leave-one-out analysis of the effect of the soluble leptin receptors on the uric acid; Figure S4. Leave-one-out analysis of the effect of the soluble leptin receptors on the gout; Table S1. The Strengthening the Reporting of Observational Studies in Epidemiology (STROBE) Statement checklist of items; Table S2. Information of databases from published genome-wide association studies (GWAS); Table S3. Characteristics of SNPs associated with adiponectin and soluble leptin receptors.

## References

[1] Rock K.L., Kataoka H., Lai J.J. (2013). Uric acid as a danger signal in gout and its comorbidities. Nat. Rev. Rheumatol..

[2] Dalbeth N., Gosling A.L., Gaffo A., Abhishek A. (2021). Gout. Lancet.

[3] Dalbeth N., Merriman T.R., Stamp L.K. (2016). Gout. Lancet.

[4] Butler F., Alghubayshi A., Roman Y. (2021). The Epidemiology and Genetics of Hyperuricemia and Gout across Major Racial Groups: A Literature Review and Population Genetics Secondary Database Analysis. J. Pers. Med..

[5] Chedid R., Zoghbi F., Halaby G., Gannage-Yared M.H. (2011). Serum uric acid in relation with the metabolic syndrome components and adiponectin levels in Lebanese University students. J. Endocrinol. Investig..

[6] Tsioufis C., Kyvelou S., Dimitriadis K., Syrseloudis D., Sideris S., Skiadas I., Katsi V., Stefanadi E., Lalos S., Mihas C. (2011). The diverse associations of uric acid with low-grade inflammation, adiponectin and arterial stiffness in never-treated hypertensives. J. Hum. Hypertens..

[7] Sirbu A.E., Buburuzan L., Kevorkian S., Martin S., Barbu C., Copaescu C., Smeu B., Fica S. (2018). Adiponectin expression in visceral adiposity is an important determinant of insulin resistance in morbid obesity. Endokrynol. Pol..

[8] Yu Y., Yang J., Fu S., Xue Y., Liang M., Xuan D., Zhu X., Wan W., Lv L., Zou H. (2019). Leptin Promotes Monosodium Urate Crystal-Induced Inflammation in Human and Murine Models of Gout. J. Immunol..

[9] Orlova I.V., Stanislavchuk M.A., Gunko I.P. (2018). Dysadipokinemia in patients with gout and its association with the disease activity. Wiadomości Lek..

[10] Oikonen M., Wendelin-Saarenhovi M., Lyytikainen L.P., Siitonen N., Loo B.M., Jula A., Seppala I., Saarikoski L., Lehtimaki T., Hutri-Kahonen N. (2012). Associations between serum uric acid and markers of subclinical atherosclerosis in young adults. The cardiovascular risk in Young Finns study. Atherosclerosis.

[11] Smith G.D., Ebrahim S. (2003). “Mendelian randomization”: Can genetic epidemiology contribute to understanding environmental determinants of disease?. Int. J. Epidemiol..

[12] Sanderson E., Davey Smith G., Windmeijer F., Bowden J. (2019). An examination of multivariable Mendelian randomization in the single-sample and two-sample summary data settings. Int. J. Epidemiol..

[13] Huffman J.E., Albrecht E., Teumer A., Mangino M., Kapur K., Johnson T., Kutalik Z., Pirastu N., Pistis G., Lopez L.M. (2015). Modulation of genetic associations with serum urate levels by body-mass-index in humans. PLoS ONE.

[14] Kottgen A., Albrecht E., Teumer A., Vitart V., Krumsiek J., Hundertmark C., Pistis G., Ruggiero D., O’Seaghdha C.M., Haller T. (2013). Genome-wide association analyses identify 18 new loci associated with serum urate concentrations. Nat. Genet..

[15] Suhre K., Arnold M., Bhagwat A.M., Cotton R.J., Engelke R., Raffler J., Sarwath H., Thareja G., Wahl A., DeLisle R.K. (2017). Connecting genetic risk to disease end points through the human blood plasma proteome. Nat. Commun..

[16] Dastani Z., Hivert M.F., Timpson N., Perry J.R., Yuan X., Scott R.A., Henneman P., Heid I.M., Kizer J.R., Lyytikainen L.P. (2012). Novel loci for adiponectin levels and their influence on type 2 diabetes and metabolic traits: A multi-ethnic meta-analysis of 45,891 individuals. PLoS Genet..

[17] Bowden J., Del Greco M.F., Minelli C., Davey Smith G., Sheehan N.A., Thompson J.R. (2016). Assessing the suitability of summary data for two-sample Mendelian randomization analyses using MR-Egger regression: The role of the I2 statistic. Int. J. Epidemiol..

[18] Hemani G., Zheng J., Elsworth B., Wade K.H., Haberland V., Baird D., Laurin C., Burgess S., Bowden J., Langdon R. (2018). The MR-Base platform supports systematic causal inference across the human phenome. Elife.

[19] Bowden J., Davey Smith G., Haycock P.C., Burgess S. (2016). Consistent Estimation in Mendelian Randomization with Some Invalid Instruments Using a Weighted Median Estimator. Genet. Epidemiol..

[20] Cheng L., Zhuang H., Ju H., Yang S., Han J., Tan R., Hu Y. (2019). Exposing the Causal Effect of Body Mass Index on the Risk of Type 2 Diabetes Mellitus: A Mendelian Randomization Study. Front. Genet..

[21] Verbanck M., Chen C.Y., Neale B., Do R. (2018). Detection of widespread horizontal pleiotropy in causal relationships inferred from Mendelian randomization between complex traits and diseases. Nat. Genet..

[22] Bowden J., Davey Smith G., Burgess S. (2015). Mendelian randomization with invalid instruments: Effect estimation and bias detection through Egger regression. Int. J. Epidemiol..

[23] Bowden J., Del Greco M.F., Minelli C., Zhao Q., Lawlor D.A., Sheehan N.A., Thompson J., Davey Smith G. (2019). Improving the accuracy of two-sample summary-data Mendelian randomization: Moving beyond the NOME assumption. Int. J. Epidemiol..

[24] Richette P., Poitou C., Manivet P., Denis J., Bouillot J.L., Clement K., Oppert J.M., Bardin T. (2016). Weight Loss, Xanthine Oxidase, and Serum Urate Levels: A Prospective Longitudinal Study of Obese Patients. Arthritis Care Res..

[25] Lyngdoh T., Vuistiner P., Marques-Vidal P., Rousson V., Waeber G., Vollenweider P., Bochud M. (2012). Serum uric acid and adiposity: Deciphering causality using a bidirectional Mendelian randomization approach. PLoS ONE.

[26] Larsson S.C., Burgess S., Michaelsson K. (2018). Genetic association between adiposity and gout: A Mendelian randomization study. Rheumatology.

[27] Inokuchi T., Tsutsumi Z., Takahashi S., Ka T., Moriwaki Y., Yamamoto T. (2010). Increased frequency of metabolic syndrome and its individual metabolic abnormalities in Japanese patients with primary gout. J. Clin. Rheumatol..

[28] Diaz-Torne C., Ortiz M.A., Garcia-Guillen A., Jeria-Navarro S., Sainz L., Fernandez-Sanchez S., Corominas H., Vidal S. (2021). The inflammatory role of silent urate crystal deposition in intercritical gout. Rheumatology.

[29] Yanai H., Adachi H., Hakoshima M., Katsuyama H. (2021). Molecular Biological and Clinical Understanding of the Pathophysiology and Treatments of Hyperuricemia and Its Association with Metabolic Syndrome, Cardiovascular Diseases and Chronic Kidney Disease. Int. J. Mol. Sci..

[30] Nishizawa T., Taniura T., Nomura S. (2015). Effects of febuxostat on platelet-derived microparticles and adiponectin in patients with hyperuricemia. Blood Coagul. Fibrinolysis.

[31] Nakata T., Ikeda S., Koga S., Yonekura T., Tsuneto A., Doi Y., Fukae S., Minami T., Kawano H., Maemura K. (2020). Randomized, Open-Label, Cross-Over Comparison of the Effects of Benzbromarone and Febuxostat on Endothelial Function in Patients with Hyperuricemia. Int. Heart J..

[32] Vitart V., Rudan I., Hayward C., Gray N.K., Floyd J., Palmer C.N., Knott S.A., Kolcic I., Polasek O., Graessler J. (2008). SLC2A9 is a newly identified urate transporter influencing serum urate concentration, urate excretion and gout. Nat. Genet..

[33] Caulfield M.J., Munroe P.B., O’Neill D., Witkowska K., Charchar F.J., Doblado M., Evans S., Eyheramendy S., Onipinla A., Howard P. (2008). SLC2A9 is a high-capacity urate transporter in humans. PLoS Med..

[34] Li S., Sanna S., Maschio A., Busonero F., Usala G., Mulas A., Lai S., Dei M., Orru M., Albai G. (2007). The GLUT9 gene is associated with serum uric acid levels in Sardinia and Chianti cohorts. PLoS Genet..

[35] Enomoto A., Kimura H., Chairoungdua A., Shigeta Y., Jutabha P., Cha S.H., Hosoyamada M., Takeda M., Sekine T., Igarashi T. (2002). Molecular identification of a renal urate anion exchanger that regulates blood urate levels. Nature.

[36] Inokuchi T., Tsutsumi Z., Takahashi S., Ka T., Yamamoto A., Moriwaki Y., Masuzaki H., Yamamoto T. (2009). Effects of benzbromarone and allopurinol on adiponectin in vivo and in vitro. Horm. Metab. Res..

[37] Miao Z., Yan S., Wang J., Wang B., Li Y., Xing X., Yuan Y., Meng D., Wang L., Gu J. (2009). Insulin resistance acts as an independent risk factor exacerbating high-purine diet induced renal injury and knee joint gouty lesions. Inflamm. Res..

[38] Doshi M., Takiue Y., Saito H., Hosoyamada M. (2011). The increased protein level of URAT1 was observed in obesity/metabolic syndrome model mice. Nucleosides Nucleotides Nucleic Acids.

[39] Lammert A., Kiess W., Bottner A., Glasow A., Kratzsch J. (2001). Soluble leptin receptor represents the main leptin binding activity in human blood. Biochem. Biophys. Res. Commun..

[40] Hirose H., Saito I., Kawai T., Tsujioka M., Kawabe H., Saruta T. (2001). Relationships between baseline serum leptin levels and 2-year changes in body mass index, blood pressure and metabolic parameters in Japanese male adolescents and middle-aged men. Clin. Sci..

[41] Lin J.D., Chiou W.K., Chang H.Y., Liu F.H., Weng H.F. (2007). Serum uric acid and leptin levels in metabolic syndrome: A quandary over the role of uric acid. Metabolism.

[42] Lyoussi B., Ragala M.A., Mguil M., Chraibi A., Israili Z.H. (2005). Gender-specific leptinemia and its relationship with some components of the metabolic syndrome in Moroccans. Clin. Exp. Hypertens.

[43] Samara A., Herbeth B., Aubert R., Berrahmoune H., Fumeron F., Siest G., Visvikis-Siest S. (2010). Sex-dependent associations of leptin with metabolic syndrome-related variables: The Stanislas study. Obesity.

[44] Aguilera A., Bajo M.A., Rebollo F., Diez J.J., Diaz C., Paiva A., Codoceo R., Selgas R. (2002). Leptin as a marker of nutrition and cardiovascular risk in peritoneal dialysis patients. Adv. Perit. Dial..

[45] Fulda S., Linseisen J., Wolfram G., Himmerich S., Gedrich K., Pollmacher T., Himmerich H. (2010). Leptin plasma levels in the general population: Influence of age, gender, body weight and medical history. Protein Pept. Lett..

[46] Obeidat A.A., Ahmad M.N., Haddad F.H., Azzeh F.S. (2016). Leptin and uric acid as predictors of metabolic syndrome in jordanian adults. Nutr. Res. Pract..

[47] Ugur-Altun B., Altun A. (2007). Circulating leptin and osteoprotegerin levels affect insulin resistance in healthy premenopausal obese women. Arch. Med. Res..

[48] Matsubara M., Chiba H., Maruoka S., Katayose S. (2002). Elevated serum leptin concentrations in women with hyperuricemia. J. Atheroscler. Thromb..

[49] Posadzy-Malaczynska A., Rajpold K., Woznicka-Leskiewicz L., Marcinkowska J. (2019). Reversal of an unfavorable effect of hydrochlorothiazide compared to angiotensin converting enzyme inhibitor on serum uric acid and oxypurine levels by estrogen-progestin therapy in hypertensive postmenopausal women. Curr. Med. Res. Opin..

[50] Koga M., Saito H., Mukai M., Kasayama S., Yamamoto T. (2009). Factors contributing to increased serum urate in postmenopausal Japanese females. Climacteric.

