# Supplementary material for: Assessing the Causal Effects of Adipokines on Uric Acid and Gout: A Two-Sample Mendelian Randomization Study
Source: Nutrients. 2022 Mar 5;14(5):1091. doi: 10.3390/nu14051091 (PMC8912555; doi:10.3390/nu14051091)
Supplement: Supplementary file 1 [file nutrients-14-01091-s001.zip › SUPPLEMENTAL MATERIAL.pdf]

Supplementary Table S1. The Strengthening the Reporting of Observational Studies in Epidemiology (STROBE) Statement checklist of items

Section/Topic	Item No	Recommendation	Manuscript section and paragraph
**Title and abstract**	1	(*a*) Indicate the study's design with a commonly used term in the title or the abstract	Title
		(*b*) Provide in the abstract an informative and balanced summary of what was done and what was found	Abstract
**Introduction**			
Background/rationale	2	Explain the scientific background and rationale for the investigation being reported	Introduction: paragraphs 1-2
Objectives	3	State specific objectives, including any prespecified hypotheses	Introduction: paragraph 3
**Methods**			
Study design	4	Present key elements of study design early in the paper	Materials and Methods
Setting	5	Describe the setting, locations, and relevant dates, including periods of recruitment, exposure, follow-up, and data collection	Materials and Methods: paragraphs 2-5
Participants	6	(*a*) *Cohort study*—Give the eligibility criteria, and the sources and methods of selection of participants. Describe methods of follow-up<br>*Case-control study*—Give the eligibility criteria, and the sources and methods of case ascertainment and control selection. Give the rationale for the choice of cases and controls<br>*Cross-sectional study*—Give the eligibility criteria, and the sources and methods of selection of participants	N/A
		(*b*) *Cohort study*—For matched studies, give matching criteria and number of exposed and unexposed<br>*Case-control study*—For matched studies, give matching criteria and the number of controls per case	N/A
Variables	7	Clearly define all outcomes, exposures, predictors, potential confounders, and effect modifiers. Give diagnostic criteria, if applicable	Materials and Methods: paragraphs 2-5
Data sources/measurement	8*	For each variable of interest, give sources of data and details of methods of assessment (measurement). Describe comparability of assessment methods if there is more than one group	Materials and Methods: paragraphs 2-5

Bias	9	Describe any efforts to address potential sources of bias	Materials and Methods: 2.4. Statistics analysis
Study size	10	Explain how the study size was arrived at	Materials and Methods: paragraphs 2-3
Quantitative variables	11	Explain how quantitative variables were handled in the analyses. If applicable, describe which groupings were chosen and why	Materials and Methods: 2.1. Datasource and selection of instruments for MR 2.4. Statistics analysis
Statistical methods	12	(*a*) Describe all statistical methods, including those used to control for confounding	Materials and Methods: 2.2. Statistics power 2.3. F-Statistics 2.4. Statistics analysis
		(*b*) Describe any methods used to examine subgroups and interactions	N/A
		(*c*) Explain how missing data were addressed	N/A
		(*d*) *Cohort study*—If applicable, explain how loss to follow-up was addressed<br>*Case-control study*—If applicable, explain how matching of cases and controls was addressed<br>*Cross-sectional study*—If applicable, describe analytical methods taking account of sampling strategy	N/A
		(*e*) Describe any sensitivity analyses	Materials and Methods: 2.4. Statistics analysis

Section/Topic	Item No	Recommendation	Manuscript section and paragraph
**Results**			
Participants	13*	(a) Report numbers of individuals at each stage of study—eg numbers potentially eligible, examined for eligibility, confirmed eligible, included in the study, completing follow-up, and analysed	N/A
		(b) Give reasons for non-participation at each stage	N/A
		(c) Consider use of a flow diagram	N/A
Descriptive data	14*	(a) Give characteristics of study participants (eg demographic, clinical, social) and information on exposures and potential confounders	N/A
		(b) Indicate number of participants with missing data for each variable of interest	N/A
		(c) *Cohort study*—Summarise follow-up time (eg, average and total amount)	N/A
Outcome data	15*	*Cohort study*—Report numbers of outcome events or summary measures over time	N/A
		Case-control study—Report numbers in each exposure category, or summary measures of exposure	N/A
		Cross-sectional study—Report numbers of outcome events or summary measures	N/A
Main results	16	(*a*) Give unadjusted estimates and, if applicable, confounder-adjusted estimates and their precision (eg, 95% confidence interval). Make clear which confounders were adjusted for and why they were included	Results: paragraphs 1-4<br>Tables 1-2<br>Figures1-4
		(*b*) Report category boundaries when continuous variables were categorized	N/A
		(*c*) If relevant, consider translating estimates of relative risk into absolute risk for a meaningful time period	N/A
Other analyses	17	Report other analyses done—eg analyses of subgroups and interactions, and sensitivity analyses	Results: paragraphs 1-4<br>Tables 1-2<br>Figures1-4<br>Supplementary Materials:<br>Supplementary Figure 1-4
**Discussion**			
Key results	18	Summarise key results with reference to study objectives	Discussion: paragraph 1
Limitations	19	Discuss limitations of the study, taking into account sources of potential bias or imprecision. Discuss both direction and magnitude of any potential bias	Discussion: paragraph 7
Interpretation	20	Give a cautious overall interpretation of results considering objectives, limitations, multiplicity of analyses, results from similar studies, and other relevant evidence	Discussion, paragraphs 2-5

Generalisability	21	Discuss the generalisability (external validity) of the study results	Discussion, paragraph 6
**Other Information**			
Funding	22	Give the source of funding and the role of the funders for the present study and, if applicable, for the original study on which the present article is based	Funding

**Give information separately for cases and controls in case-control studies and, if applicable, for exposed and unexposed groups in cohort and cross-sectional studies.*

Note: An Explanation and Elaboration article discusses each checklist item and gives methodological background and published examples of transparent reporting. The STROBE checklist is best used in conjunction with this article (freely available on the Web sites of PLoS Medicine at http://www.plosmedicine.org/, Annals of Internal Medicine at http://www.annals.org/, and Epidemiology at http://www.epidem.com/). Information on the STROBE Initiative is available at www.strobe-statement.org.

Supplementary Table S2. Information of databases from published genome-wide association studies (GWAS)

Database	Authors	Sample size	Ancestry	Phenotype measurement	The units
UA	Huffman, JE. et al	42,741	European	Uricase method	mg/dl
Gout	Kottgen, A. et al	69,374	European	Based on self-report and medication	N/A
Adiponectin	Dastani, Z.et al	45,891	Predominantly European	ELISA or RIA methods	μg/ml
sOB-R	Suhre, K. et al	1,000	European	Ultrahigh performance liquid-phase chromatography and gas chromatography separation coupled with tandem mass spectrometry	N/A

Supplementary Table S3. Characteristics of SNPs associated with adiponectin and soluble leptin receptors

SNPs	Genomic Coordinate	EA/OA	β	SE	*P*-value	MAF	Mean F-statistic
**adiponectin *vs.* uric acid**							
rs17366568	Chr3:186570453	A/G	-0.154	0.009	1.00E-200	0.908	292.8
rs3774261	Chr3:186571559	G/A	-0.070	0.005	1.00E-200	0.400	196.0
rs6810075	Chr3:186548565	C/T	-0.066	0.005	1.00E-200	0.633	174.2
rs6488898	Chr12:124203832	A/G	0.054	0.009	1.48E-09	0.950	36.0
rs731839	Chr19:33899065	A/G	0.037	0.005	2.20E-13	0.672	54.8
rs864265	Chr3:186554292	G/T	0.037	0.006	1.43E-08	0.875	38.0
rs7955516	Chr12:20498036	C/A	0.026	0.005	2.43E-08	0.442	27.0
rs1108842	Chr3:52720080	C/A	0.030	0.004	3.66E-11	0.458	56.3
rs2925979	Chr16:81534790	C/T	0.044	0.005	1.87E-18	0.717	77.4

rs1426810	Chr3:186503435	A/G	-0.054	0.005	2.84E-30	0.625	116.6
rs7200895	Chr16:82644606	T/C	0.044	0.006	1.85E-12	0.150	53.8
rs1870843	Chr16:82759314	A/G	-0.032	0.005	4.26E-11	0.608	41.0
rs863750	Chr12:124505444	T/C	-0.027	0.005	6.41E-09	0.467	29.2
rs11057405	Chr12:122781897	A/G	-0.052	0.009	5.58E-09	0.908	33.4
rs7133378	Chr12:124409502	A/G	0.030	0.005	1.29E-09	0.627	36.0
rs1597466	Chr3:150055561	T/G	-0.044	0.008	1.89E-08	0.092	30.3
rs5030072	Chr3:186455546	C/T	0.030	0.005	9.89E-11	0.573	36
rs601339	Chr12:123174743	G/A	0.039	0.006	3.87E-11	0.150	42.3
rs8058318	Chr16:82628245	G/A	0.028	0.005	3.26E-08	0.317	31.4
rs822387	Chr3:186556037	C/T	0.148	0.009	1.00E-200	0.900	270.4
rs11865200	Chr16:82625705	G/A	-0.040	0.006	1.14E-09	0.170	44.4
rs822354	Chr3:186480206	G/A	-0.050	0.005	1.53E-20	0.333	100
rs2062632	Chr3:186461181	C/T	-0.055	0.006	2.52E-19	0.686	84.0
**adiponectin *vs.* gout**							
rs17366568	Chr3:186570453	A/G	-0.154	0.009	1.00E-200	0.908	292.8
rs3774261	Chr3:186571559	G/A	-0.070	0.005	1.00E-200	0.400	196.0
rs6810075	Chr3:186548565	C/T	-0.066	0.005	1.00E-200	0.633	174.2
rs6488898	Chr12:124203832	A/G	0.054	0.009	1.48E-09	0.950	36.0
rs731839	Chr19:33899065	A/G	0.037	0.005	2.20E-13	0.672	54.8
rs864265	Chr3:186554292	G/T	0.037	0.006	1.43E-08	0.875	38.0
rs7955516	Chr12:20498036	C/A	0.026	0.005	2.43E-08	0.442	27.0
rs1108842	Chr3:52720080	C/A	0.030	0.004	3.66E-11	0.458	56.3
rs2925979	Chr16:81534790	C/T	0.044	0.005	1.87E-18	0.717	77.4
rs12051272	Chr16:82663288	T/G	-0.277	0.018	1.00E-200	0.009	236.8
rs1426810	Chr3:186503435	A/G	-0.054	0.005	2.84E-30	0.625	116.6

rs7200895	Chr16:82644606	T/C	0.044	0.006	1.85E-12	0.150	53.8
rs1870843	Chr16:82759314	A/G	-0.032	0.005	4.26E-11	0.608	41.0
rs863750	Chr12:124505444	T/C	-0.027	0.005	6.41E-09	0.467	29.2
rs8042532	Chr15:74255230	G/T	-0.340	0.055	2.86E-09	0.992	38.2
rs11057405	Chr12:122781897	A/G	-0.052	0.009	5.58E-09	0.908	33.4
rs7133378	Chr12:124409502	A/G	0.030	0.005	1.29E-09	0.627	36.0
rs1597466	Chr3:150055561	T/G	-0.044	0.008	1.89E-08	0.092	30.3
rs5030072	Chr3:186455546	C/T	0.030	0.005	9.89E-11	0.573	36
rs601339	Chr12:123174743	G/A	0.039	0.006	3.87E-11	0.150	42.3
rs8058318	Chr16:82628245	G/A	0.028	0.005	3.26E-08	0.317	31.4
rs822387	Chr3:186556037	C/T	0.148	0.009	1.00E-200	0.900	270.4
rs11865200	Chr16:82625705	G/A	-0.040	0.006	1.14E-09	0.170	44.4
rs822354	Chr3:186480206	G/A	-0.050	0.005	1.53E-20	0.333	100
rs2062632	Chr3:186461181	C/T	-0.055	0.006	2.52E-19	0.686	84.0
**soluble leptin receptors *vs.* uric acid**							
rs11208654	Chr1:65965566	C/T	-0.317	0.046	7.90E-12	N/A	47.5
rs7535099	Chr1:66050997	G/A	0.346	0.051	3.06E-11	N/A	46.0
rs4655537	Chr1:66058801	G/A	-0.350	0.044	7.42E-15	N/A	63.3
rs17415296	Chr1:66099013	A/C	-1.400	0.032	1.00E-200	N/A	1, 914.1
**soluble leptin receptors *vs.* gout**							
rs11208654	Chr1:65965566	C/T	-0.317	0.046	7.90E-12	N/A	47.5
rs7535099	Chr1:66050997	G/A	0.346	0.051	3.06E-11	N/A	46.0
rs4655537	Chr1:66058801	G/A	-0.350	0.044	7.42E-15	N/A	63.3
rs17415296	Chr1:66099013	A/C	-1.400	0.032	1.00E-200	N/A	1, 914.1

SNP: single-nucleotide polymorphism; OA: other allele; SE: standard error; MAF: minor allele frequency.
