# Supplementary figures and images for: Assessing the Causal Effects of Adipokines on Uric Acid and Gout: A Two-Sample Mendelian Randomization Study
Source: Nutrients. 2022 Mar 5;14(5):1091. doi: 10.3390/nu14051091 (PMC8912555; doi:10.3390/nu14051091)

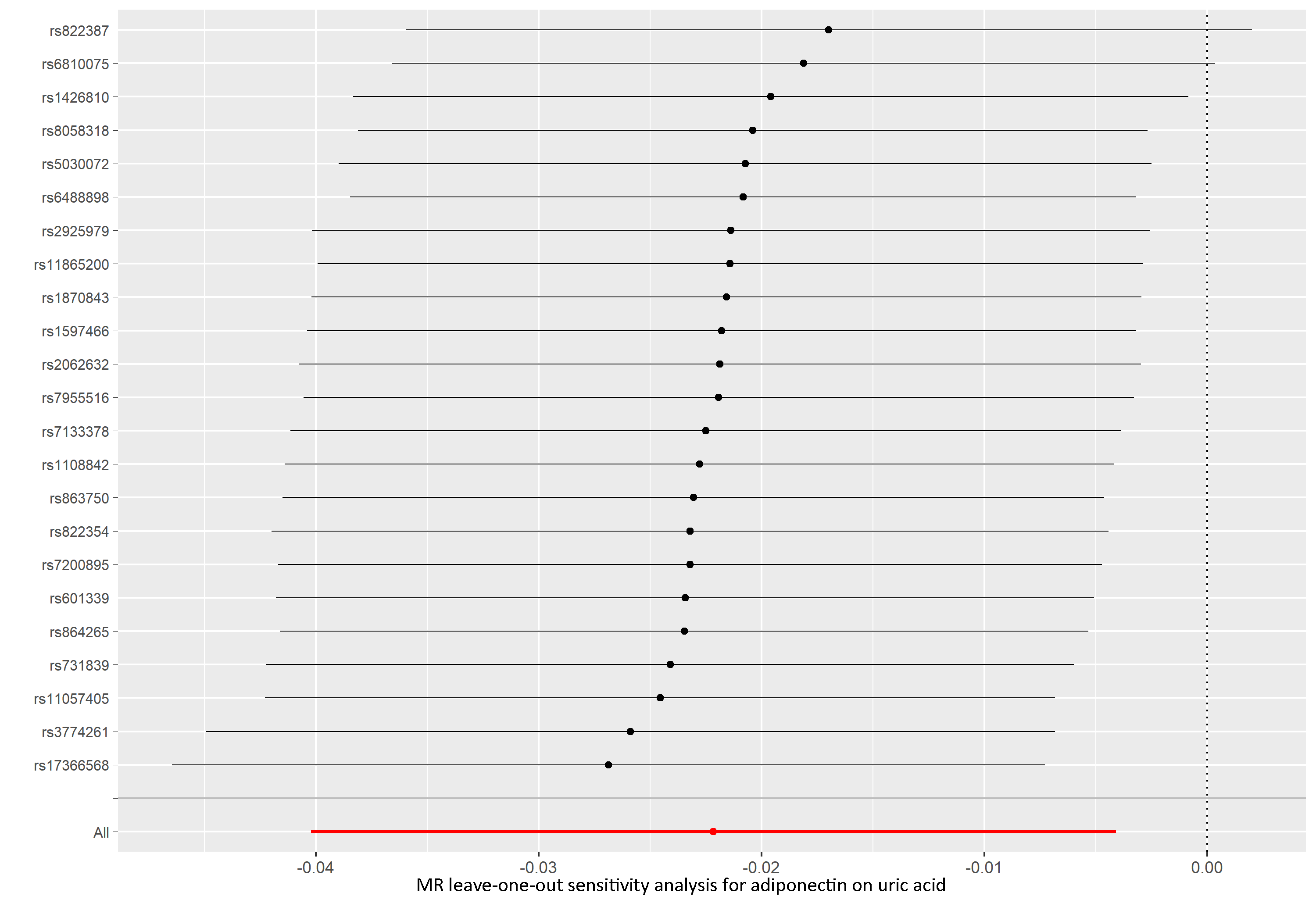

Supplement: Supplementary file 1 [file nutrients-14-01091-s001.zip › Supplementary Figure S1.tiff]

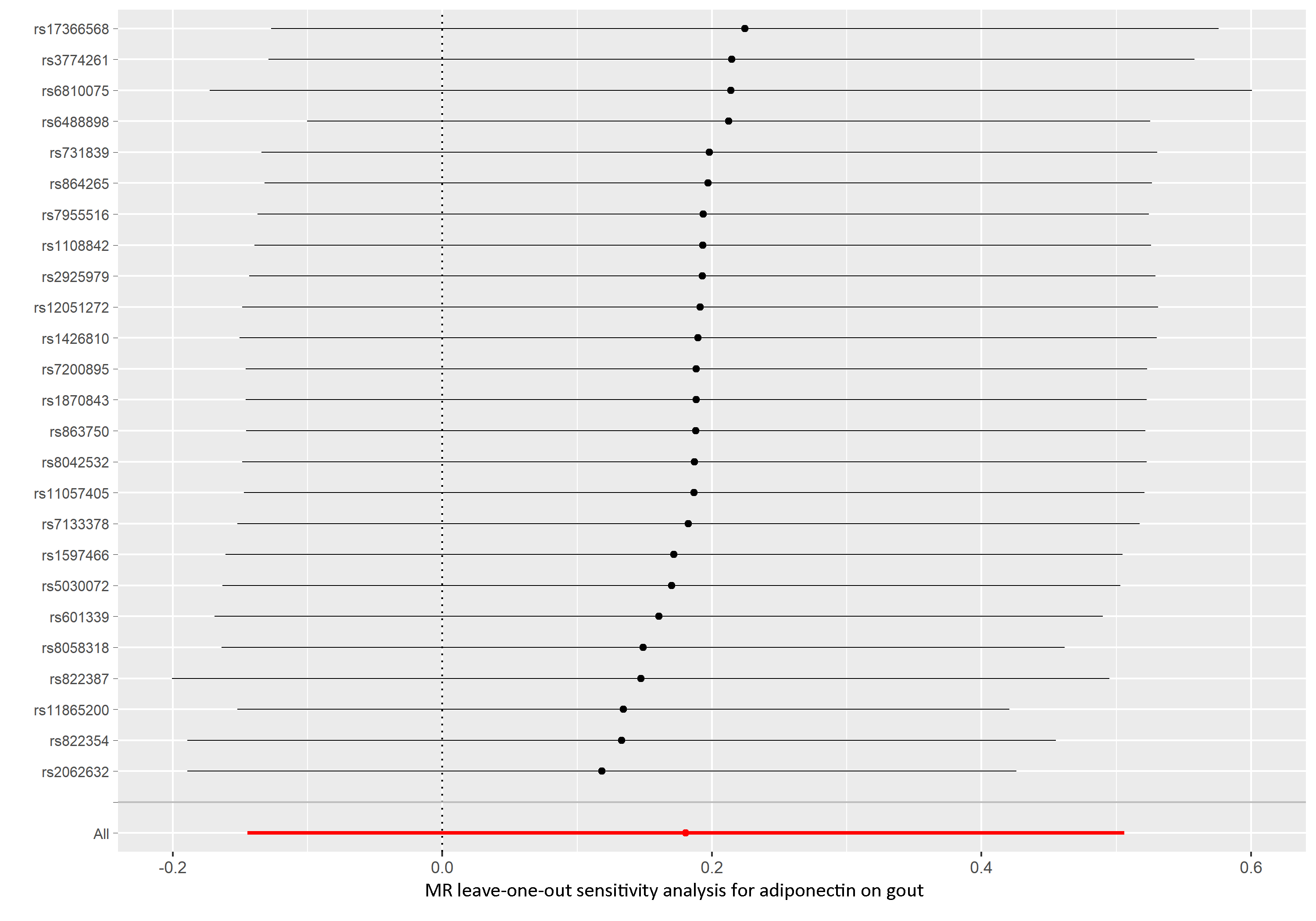

Supplement: Supplementary file 1 [file nutrients-14-01091-s001.zip › Supplementary Figure S2.tif]

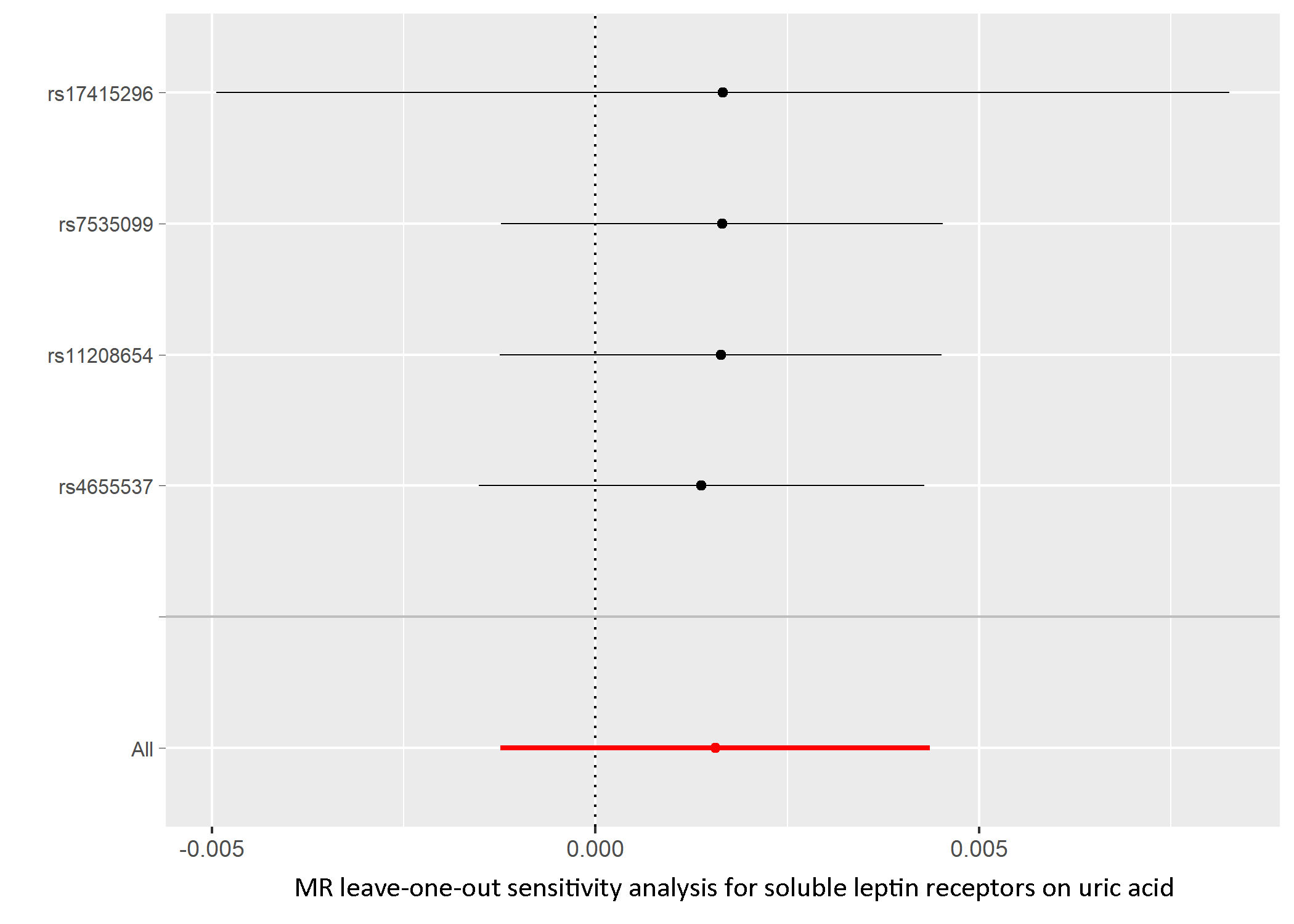

Supplement: Supplementary file 1 [file nutrients-14-01091-s001.zip › Supplementary Figure S3.tif]

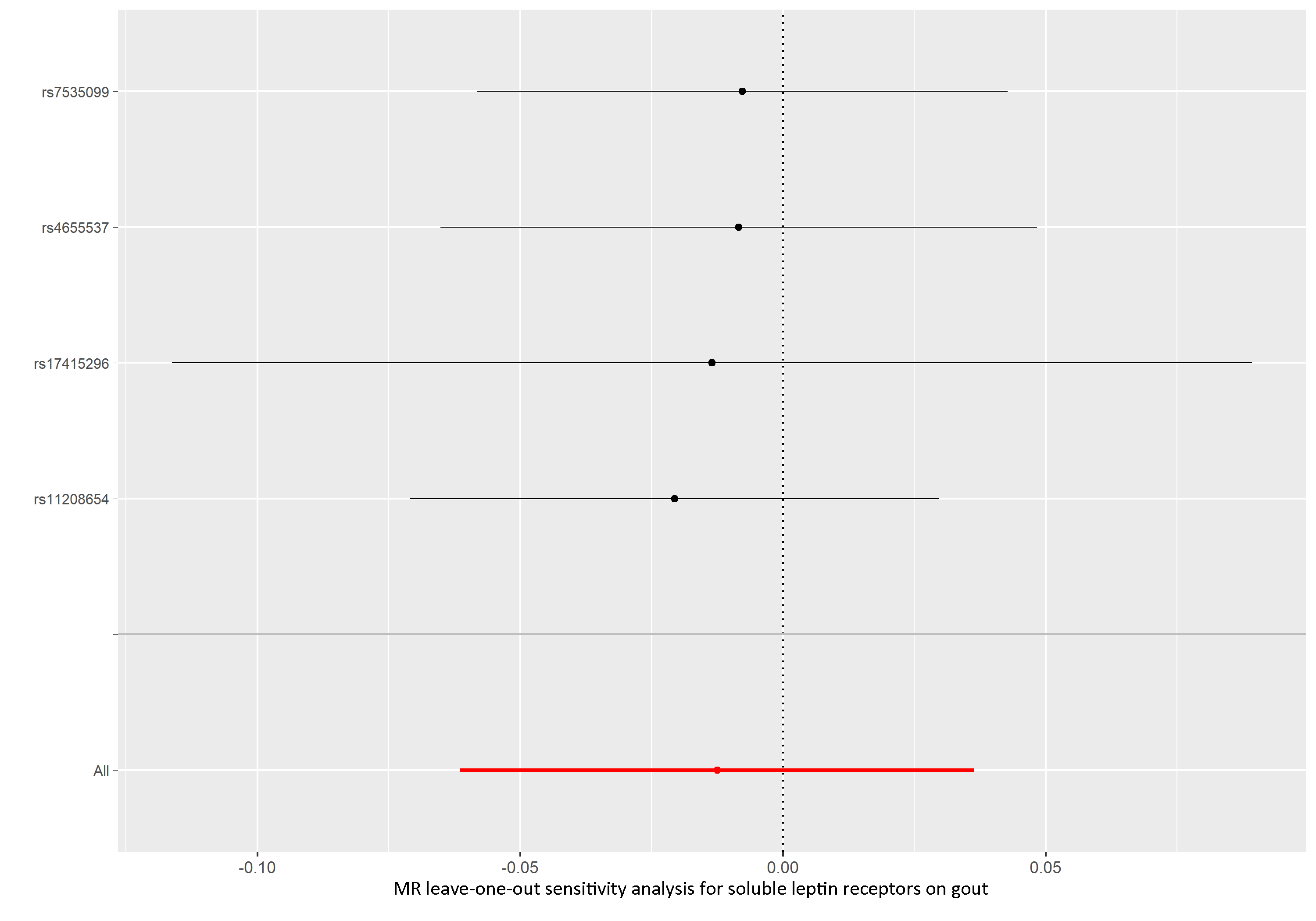

Supplement: Supplementary file 1 [file nutrients-14-01091-s001.zip › Supplementary Figure S4.tif]
